# *LIMCH1* in Cancer: A Context-Dependent Modulator Beyond the Tumor Suppressor–Oncogene Dichotomy

**DOI:** 10.3390/ijms27188144

**Published:** 2026-09-12

**Authors:** Mengying Guan, Hua Hao

**Affiliations:** 1 Department of Breast Surgery, Yangpu Hospital, School of Medicine, Tongji University, Shanghai 200090, China; 2480166@tongji.edu.cn; 2 Department of Pathology, Yangpu Hospital, School of Medicine, Tongji University, Shanghai 200090, China

**Keywords:** *LIMCH1*, tumor suppressor, oncogene, context-dependent regulation, actomyosin contractility

## Abstract

LIM and calponin homology domain-containing protein 1 (*LIMCH1*) has recently gained attention as a functionally perplexing factor in human cancers, with studies attributing both tumor-suppressive and tumor-promoting properties that defy simple categorization. Originally described as an actin-associated cytoskeletal protein that promotes non-muscle myosin-IIA (NM-IIA) activity and thereby curtails cell migration, *LIMCH1* has more recently been linked to diverse oncogenic pathways across a range of tumor types. In lung adenocarcinoma and clear-cell renal cell carcinoma, diminished *LIMCH1* expression is associated with more aggressive clinical behavior and reduced overall survival, aligning with a tumor-suppressive role. By comparison, in triple-negative and invasive breast cancers and in cervical squamous cell carcinoma, increased *LIMCH1* expression correlates with enhanced metastatic spread, immune escape, and unfavorable outcomes, suggesting a pro-oncogenic function. In this review, findings from over 30 published studies are integrated with an original TCGA pan-cancer expression and survival analysis to propose that *LIMCH1* is best understood as a context-dependent conditional regulator rather than as a classical tumor suppressor or oncogene. Based on the available evidence, we propose a hypothesis-generating conceptual framework in which its ultimate phenotypic effect is shaped by three interacting contextual determinants: (i) *TP53* mutation status (determining whether the *HUWE1*-p53 axis is intact); (ii) tumor matrix stiffness (modulating actomyosin contractility and migration mode); and (iii) upstream signaling cues (e.g., MAPK/ERK, TGFβ). Of note, other tumor-lineage and microenvironmental factors may also contribute and warrant further investigation. This framework resolves the apparent functional duality of *LIMCH1* in a cancer type-specific manner and provides critical guidance for future mechanistic research, standardized detection workflows, and the clinical translation of *LIMCH1*-based biomarkers.

## 1. Introduction

Cancer biology has repeatedly identified proteins that do not align neatly with the traditional classification of tumor suppressors versus oncogenes. LIM and calponin homology domain-containing protein 1 (*LIMCH1*, also known as *KIAA1102*) is a particularly illustrative case. First identified through co-immunoprecipitation and mass spectrometry as a direct interactor of non-muscle myosin-IIA (NM-IIA), *LIMCH1* was initially characterized as a regulator of actin stress fiber organization and contractility [1]. Since then, *LIMCH1* has been implicated in a wide spectrum of malignancies—including lung [2], breast [3,4,5], renal [6,7,8], cervical [9], esophageal [10], ovarian [11], endometrial [12,13], thyroid [14], and oral cancers [15]—where it exhibits roles that defy simple categorization. In some contexts, it stabilizes p53 and suppresses cell proliferation [16]; in others, it drives immune evasion by inducing PD-L1 and supports metastatic spread [3]. This bidirectional functionality has immediate therapeutic ramifications: whether *LIMCH1* should be pharmacologically boosted or inhibited is likely to depend on the cancer type and biological setting.

While multiple studies have examined *LIMCH1* expression in specific tumors, none has comprehensively addressed the mechanistic inconsistencies that characterize the current literature. To our knowledge, this review offers an integrated synthesis that: combines cytoskeletal mechanobiology, p53 signaling, and immune-oncology into a unified conceptual framework that reconciles *LIMCH1*’s functional duality; performs a standardized pan-cancer re-analysis of raw TCGA STAR-Counts using DESeq2 to resolve pipeline-driven discrepancies; and systematically dissects methodological sources of conflicting observations—including antibody isoform specificity and heterogeneous quantification pipelines. Small cohort sizes, heterogeneous antibody reagents, and isoform-specific expression have all contributed to conflicting reports. Compounding this, many studies have relied on limited bioinformatic approaches without subsequent mechanistic follow-up, leading to findings in which *LIMCH1* is reported as both upregulated and downregulated [2,3,4,9]. This review directly tackles these contradictions. The molecular structure and canonical biological functions of *LIMCH1* are first characterized; then, the preclinical and clinical evidence for its tumor-suppressive and pro-tumorigenic roles are separately integrated. The methodological drivers of conflicting observations across studies are next dissected, followed by an original pan-cancer analysis of *LIMCH1* expression and prognostic value across TCGA tumor types. Based on these findings, a unified context-dependent regulatory model is proposed to resolve *LIMCH1*’s functional duality, and, finally, the clinical translational implications and key unresolved questions for future research are discussed.

Literature search and selection. We searched PubMed, Web of Science Core Collection and Google Scholar from database inception to the final update before submission using combinations of “*LIMCH1*”, “*KIAA1102*”, “cancer”, “tumor”, “oncogene”, “tumor suppressor”, “prognosis”, “metastasis”, “EMT”, “actomyosin” and “non-muscle myosin II”, with citation tracking and hand-searching of reference lists. Eligible records were English-language original research articles or reviews reporting molecular, functional, clinical or transcriptomic data on *LIMCH1* in human cancer or relevant mechanistic models; conference abstracts, editorials, records without accessible full text and studies without cancer-related or mechanistic endpoints were excluded.

## 2. LIMCH1 Gene and Protein: Structure and General Function

*LIMCH1* is a member of the LIM/CH domain superfamily—modular cytoskeletal proteins defined by zinc-binding LIM domains that mediate protein–protein interactions and CH domains that associate with actin filaments [17]. In a seminal study, Lin et al. [1] demonstrated through co-immunoprecipitation and mass spectrometry that *LIMCH1* forms a stable complex with the heavy chain of NM-IIA (*MYH9*). GST pull-down experiments localized this interaction to the N-terminal coiled-coil region. The N-terminal CH domain is presumed to bind actin based on the canonical role of CH domains, whereas the C-terminal LIM domain mediates protein–protein interactions. Together, these features define a tripartite assembly in which *LIMCH1* functions as a structural adaptor that regulates actomyosin contractility.

The functional landscape is further complicated by extensive isoform diversity. Friedberg et al. [17] predicted multiple *LIMCH1* splice variants with variable domain retention; Penna et al. [18] subsequently validated tissue-specific variants in skeletal muscle. These findings raise the possibility that cancer cells may express tumor-specific isoforms with divergent functions. Critically, no study has systematically profiled *LIMCH1* splice variant distribution across cancer types, nor has any study identified which isoforms are functionally dominant in specific tumor contexts. This gap directly affects the interpretation of published studies, as different antibodies may preferentially recognize distinct isoforms [17,18]. Isoform-aware analysis must, therefore, be a prerequisite for future investigations of *LIMCH1* in cancer.

In addition to its cytoskeletal functions, the CH-LIM family proteins LMO7 and PDLIM1 contribute to the actin-dependent recruitment of AGO2 to the zonula adherens in cooperation with PLEKHA7 [19]; whether *LIMCH1* itself participates in this complex has not been tested. This places the *LIMCH1*/LMO7 scaffold family at the intersection of cytoskeletal dynamics and post-transcriptional gene regulation. *LIMCH1* has also been implicated in glucocorticoid-responsive signaling, with upregulated expression observed in dexamethasone-treated trabecular meshwork cells undergoing Wnt/planar cell polarity (PCP)-dependent actin reorganization [20].

Nevertheless, *LIMCH1* remains fundamentally a key regulator of NM-IIA: Feroz et al. [21] listed *LIMCH1* among the binding partners that govern NM-IIA filament assembly and function. Given the central importance of NM-IIA and integrin-mediated adhesion in cell migration, *LIMCH1* emerges as a critical upstream regulator of actomyosin contractility [22,23].

## 3. Evidence for Tumor-Suppressive Roles

### 3.1. LIMCH1 as a Cytoskeletal Modulator

The most robust and mechanistically reproducible support for a tumor-suppressive role of *LIMCH1* derives from its function in cytoskeletal organization. Functional follow-up by Lin et al. [1] showed that *LIMCH1* knockdown in HeLa cells decreases diphosphorylation of myosin regulatory light chain (MRLC), reduces the abundance of stress fibers and focal adhesions, and increases both two-dimensional migration rates and collagen-gel contraction; reintroduction of an siRNA-resistant wild-type *LIMCH1* construct reversed these effects, confirming the specificity of the knockdown phenotype. These findings establish the core mechanism: *LIMCH1* restricts cell migration by sustaining NM-IIA-dependent actomyosin contractility, thereby maintaining an actin architecture that resists the cytoskeletal remodeling required for malignant invasion.

From this established *LIMCH1*–NM-IIA axis, two convergent mechanistic routes for tumor suppression can be logically inferred: one operating through adherens-junction mechanotransduction, and another through the regulation of migration mode switching.

First, an adherens-junction (AJ) mechanotransduction route is discussed. At epithelial junctions, NM-IIA links the E-cadherin–catenin complex to the perijunctional actin belt and generates the tension that stabilizes the zonula adherens [24,25]. Betapudi [26] further revealed that distinct functional classes of myosin II motor proteins differentially govern lamellipodia extension during cell spreading, underscoring that the mechanical output of the actomyosin cortex is finely tuned by motor protein activity. Because LIMCH1 binds the N-terminal coiled-coil region of the NM-IIA heavy chain and is required to sustain MRLC phosphorylation and stress-fiber integrity [1], its loss would logically lower perijunctional tension, weaken adherens junctions, and thereby release junctional control over β-catenin and EGFR localization—a plausible but experimentally untested route from *LIMCH1* downregulation to EMT initiation. Although not directly demonstrated for *LIMCH1*, the established architecture of adherens junctions suggests that NM-IIA-generated contractile forces are sensed by αE-catenin, whose actin-binding domain forms a direction-dependent catch bond with F-actin [27,28,29,30]. In a broader migratory context, Vassilev et al. [31] demonstrated that catenins steer cell migration via the stabilization of front-rear polarity, implying that αE-catenin and its associated proteins are not merely passive mechanosensors but active determinants of migration directionality. Junctional recruitment assays, αE-catenin tension biosensors, and EGFR-localization measurements in *LIMCH1*-manipulated epithelial cells would provide direct tests of this hypothesis.

Beyond stabilizing cell–cell junctions, NM-IIA contractility also governs how cells interact with the extracellular matrix and navigate through physically confined microenvironments. Second, a route involving mesenchymal-to-amoeboid migration mode switching is discussed. The Rho–ROCK–myosin axis constitutes the main control node that converts physical substrate stiffness into intracellular biochemical commands [32]. From a mechanobiological perspective, Bergert et al. [33] demonstrated that effective force transmission during adhesion-independent migration critically depends on actin–myosin contractility. Because *LIMCH1* directly tunes NM-IIA contractile activity, it occupies a key position in governing the switch between mesenchymal and amoeboid migration modes. In matrix-rich microenvironments, such as the lung parenchyma, loss of *LIMCH1* would be expected to favor mesenchymal, protease-driven invasion; in confined spaces, such as lymphatic vessels or dense interstitial matrix, sustained *LIMCH1* expression may shift cells toward contractility-driven, rapid amoeboid motility [34,35]. Notably, Singh et al. [36] showed that NM-IIA regulates aortic stiffness through effects on specific focal adhesion proteins and the non-muscle cortical cytoskeleton, establishing a broader principle that NM-IIA-mediated contractility is a central hub in cell–matrix mechanosensing across diverse tissue contexts. Thus, through its modulation of NM-IIA contractility, *LIMCH1* serves as a critical determinant of which migration strategy cells adopt in a given microenvironment.

Indirect supportive evidence is obtained from breast cancer models, where Bersini et al. [37] observed that *LIMCH1* upregulation upon *NUP93* depletion correlates with reduced invasive behavior, consistent with a migration-restraining function for *LIMCH1*. This observation also illustrates a recurring theme in the *LIMCH1* literature: its functional output is dictated by experimental context—cell model, assay readout and immune milieu—rather than by tumor type alone, as the same anti-invasive effect of *LIMCH1* in a triple-negative breast cancer model contrasts with the pro-metastatic, immune-evasive functions described in other breast cancer settings [3,5]. Nevertheless, whether *LIMCH1* upregulation is the direct causal effector in the *NUP93* depletion setting has not been independently validated; this evidence should, therefore, be interpreted as correlational support rather than definitive proof.

Beyond its NM-IIA-dependent cytoskeletal functions, *LIMCH1* may also modulate signaling through an NM-IIA-independent route. Karlsson et al. [38] reported that *LIMCH1*—like its homolog LMO7 [39]—directly interacts with the transmembrane proteins LRIG1 and LRIG3, established negative regulators of receptor tyrosine kinases, including EGFR, and that these interactions carry prognostic significance in early-stage lung cancer. This points to a parallel pathway through which *LIMCH1* may influence RTK/EGFR signaling independently of its effects on actomyosin contractility, although the functional consequences of this interaction in cancer cells remain to be fully characterized.

### 3.2. Lung Adenocarcinoma: The p53–HUWE1 Axis and Clinical Correlates

Zhang et al. [16] characterized a specific tumor-suppressive mechanism in A549 lung adenocarcinoma cells. Using co-immunoprecipitation, ubiquitination assays, and flow cytometry, they showed that *LIMCH1* interacts with the E3 ubiquitin ligase *HUWE1*, which ordinarily promotes proteasomal degradation of p53. By binding to *HUWE1*, *LIMCH1* sterically blocked the *HUWE1*–p53 interaction, thereby preserving p53 protein levels. As a result, *LIMCH1* overexpression in A549 cells triggered G0/G1 cell-cycle arrest and reduced clonogenic growth. Separately, Yang et al. [40] confirmed that *HUWE1* drives NSCLC progression through p53 suppression. Gong et al. [41] provided a comprehensive review of *HUWE1* structure, regulation, and oncogenic functions, supplying a crucial background for understanding the *LIMCH1*–*HUWE1*–p53 axis.

Beyond this mechanistic evidence, clinical data support a tumor-suppressive role for *LIMCH1* in lung adenocarcinoma. Cao et al. [2] examined 196 resected lung adenocarcinoma specimens by tissue microarray immunohistochemistry using the Abcam (Cambridge, UK) ab96178 polyclonal antibody. *LIMCH1* protein expression was significantly decreased in tumors compared with the matched adjacent normal lung; low expression correlated with pleural invasion, larger tumor size, poorer differentiation, and more advanced clinical stage. Kaplan–Meier analysis demonstrated notably shorter overall survival in patients with low *LIMCH1* expression; multivariate Cox regression identified *LIMCH1* as an independent favorable prognostic factor (HR = 0.41, 95% CI 0.22–0.75, *p* = 0.004). In addition, gene set enrichment analysis (GSEA) of TCGA datasets showed that tumors with low *LIMCH1* expression were enriched for mTORC1 signaling, *MYC* target gene sets, DNA repair pathways, and G2–M checkpoint programs.

The parallel reduction in protein levels, their correlation with unfavorable pathological characteristics, poorer patient survival, and enrichment of oncogenic pathways together argue that *LIMCH1* likely functions as a tumor suppressor in lung cancer. Nonetheless, some caution is appropriate: the study by Cao et al. was conducted at a single Chinese institution; although their antibody validation was internally coherent, it did not include comparison with recombinant *LIMCH1* standards or independent verification by mass spectrometry. Of note, the same Abcam (Cambridge, UK) ab96178 antibody and similar TCGA-based analytical approaches have produced strikingly opposite conclusions in triple-negative breast cancer, where higher *LIMCH1* expression is associated with worse, rather than better, clinical outcomes [3]; potential explanations for this conflict are discussed in Section 7 (Competing Molecular Circuits: A Context-Dependent Regulatory Model).

Single-cell data from lung adenocarcinoma also point toward a protective role. *LIMCH1* emerged as one of eight prognostic genes in a cancer-associated fibroblast signature tied to epithelial–mesenchymal transition and carried a negative risk coefficient [42]. This indicates that higher expression predicted a better outcome in that particular classifier.

### 3.3. Renal Cell Carcinoma, Non-Coding RNA Networks, and Other Malignancies

Clear-cell renal cell carcinoma (ccRCC) offers a similarly illustrative protective example. In an Affymetrix microarray study examining smoking-associated ccRCC, Eckel-Passow et al. [6] identified *LIMCH1* among genes with smoking-related expression changes, noting its upregulation in tumors from smokers relative to nonsmokers; however, this association did not reach significance in subsequent RT-PCR validation. Prognostic models generated by Yao et al. [7] (using bioinformatic analyses of public datasets) and Terrematte et al. [8] included *LIMCH1* as part of an NK cell–related signature and a 13-gene panel, respectively, with both reports linking higher *LIMCH1* expression to more favorable clinical outcomes. The 13-gene signature by Terrematte et al. was derived through machine learning-based feature selection followed by LASSO Cox regression. Its predictive performance was internally validated by bootstrap resampling; however, independent prospective validation remains pending.

Non-coding RNAs modulate *LIMCH1* expression across multiple cancer types. In nonsquamous NSCLC, Wang et al. [43] proposed a competing endogenous RNA (ceRNA) circuit in which *circLDB2* binds *miR-346*, thereby alleviating its repression of *LIMCH1* translation; consequent *LIMCH1* elevation restrained tumor progression and increased cisplatin responsiveness. Similarly, Zu et al. [44] identified *miR-660-5p* as a metastasis-promoting miRNA that directly targets the *LIMCH1* 3′-UTR in NSCLC: *miR-660-5p*–mediated silencing of *LIMCH1* enhanced multi-organ metastatic colonization, whereas restoration of *LIMCH1* reversed the phenotype. A related ccRCC ceRNA study reported *circPPAP2B*-dependent control of metastasis [45], although *LIMCH1* was not the direct effector assayed in that work, limiting the mechanistic inference that can be drawn from this observation. Beyond these specific ceRNA circuits, Grzywa et al. [46] systematically reviewed how microRNAs broadly drive tumor cell invasiveness and metastasis by targeting cytoskeletal regulators and migration-related genes, suggesting that *LIMCH1* may integrate into wider post-transcriptional control networks governing cell motility.

The tumor-suppressive framework extends beyond renal cancer to head and neck malignancies. Li et al. [15] demonstrated that *LIMCH1* is substantially downregulated in oral squamous cell carcinoma relative to matched normal mucosa, with TCGA data supporting this reduction. Functional assays further revealed that *LIMCH1* overexpression in Cal27 OSCC cells suppressed migration and proliferation while promoting apoptosis, suggesting a protective role in oral tumorigenesis, although the underlying molecular mechanisms warrant further investigation. An additional perspective is provided by a small GWAS in oral verrucous hyperplasia cohorts, which identified seven genome-wide significant loci for malignant conversion, including the intronic SNP rs75857466 within *LIMCH1* [47]. The effect size is modest and the mechanism is unknown, but the fact that an unbiased screen picked up this locus raises the possibility that inherited differences in *LIMCH1* modulate susceptibility to oral carcinogenesis.

Collectively, the tumor-suppressive roles reported in renal and oral cancers are based primarily on in vitro experiments and clinical association studies; definitive causal validation from in vivo gene-editing models in these cancer types is still missing.

## 4. Evidence for Pro-Tumorigenic Roles

### 4.1. Triple-Negative Breast Cancer: Immune Evasion via the MAPK–PD-L1 Axis

Although studies in lung and kidney cancers support a tumor-suppressive role for *LIMCH1*, reports in breast and cervical cancers largely conflict with this conclusion. Recent studies have provided critical new mechanistic and clinical insights into *LIMCH1*’s oncogenic role in breast cancer. Qiu et al. [3] performed the most extensive functional characterization of *LIMCH1* in any cancer type to date, concentrating on triple-negative breast cancer (TNBC). In their 2025 study, combining a 4T1 syngeneic allograft model with clinical cohort validation, they firmly linked *LIMCH1* to MAPK/ERK signaling, PD-L1 upregulation, and CD4+/CD8+ T-cell exclusion, thereby establishing *LIMCH1* as a driver of immunosuppressive breast cancer microenvironments. These findings were independently supported by Yu et al. [5], who used integrative bioinformatics and immunohistochemistry to connect high *LIMCH1* expression to immunosuppressive infiltration patterns and unfavorable prognosis. Using TCGA pan-cancer RNA-seq data, a breast cancer tissue microarray stained with Abcam (Cambridge, UK) ab96178 (1:500), and the murine TNBC cell lines 4T1 and EMT6, they demonstrated that both *LIMCH1* mRNA and protein are elevated in TNBC relative to normal breast tissue and luminal breast cancer subtypes. The mechanistic underpinnings of this seeming discrepancy—how the same protein can act as a protective factor in lung adenocarcinoma yet promote disease in TNBC—are examined in the context-dependent model presented in Section 7, with p53 mutation status and biomechanical signals highlighted as major determinants.

Mechanistically, Qiu et al. [3] (combining a 4T1 syngeneic allograft model with validation in clinical cohorts) attributed *LIMCH1*’s pro-tumorigenic effects to activation of the MAPK pathway. RNA-seq of *LIMCH1*-overexpressing 4T1 cells identified MAPK/ERK signaling as the most prominently affected pathway. Western blot and immunofluorescence assays revealed that *LIMCH1*-overexpressing 4T1 cells display markedly increased PD-L1 levels at the cell surface, a change mediated by the AP-1 components c-Jun and ATF3 and abrogated by the MEK inhibitor trametinib. Flow cytometric analysis of tumor-infiltrating lymphocytes from BALB/c syngeneic allografts (approved by the Nankai University ethics committee, SHYJS-CP-1910006) indicated reduced CD4+ and CD8+ T-cell subsets in *LIMCH1*-high tumors; multiplex immunohistochemistry confirmed that regions with strong *LIMCH1* staining were relatively deficient in T-cell infiltration. In vivo, enforced *LIMCH1* expression accelerated primary tumor expansion and increased the burden of metastatic lung nodules.

Collectively, these findings indicate that *LIMCH1* helps TNBC tumors create an immunologically cold microenvironment while simultaneously driving metastatic spread [3]. Notably, high *LIMCH1* expression also tracked with poor anti-PD-1 response in silico. If this pattern is prospectively validated, *LIMCH1* would behave unlike typical PD-L1 biomarkers. Rather than identifying immunologically active tumors likely to respond to checkpoint blockade, it might instead mark cold, excluded tumors where immunotherapy is unlikely to work. This interpretation remains speculative, but if confirmed, it could have significant translational potential.

Glycosyltransferase-based molecular subtyping adds supporting evidence. In the aggressive C2 breast cancer subtype, *LIMCH1* emerged as one of four central hub genes [48]. Its expression moved inversely with cytotoxic CD8+ T-cell density and in the same direction as M2 macrophage accumulation. These immune correlations suggest that LIMCH1 may construct an immunosuppressive niche that converges on the same endpoint as the MAPK–PD-L1 axis, albeit via a distinct molecular route.

### 4.2. Breast Cancer: Metastasis and the Amoeboid Switch

Alifanov et al. [4] extended these findings by linking *LIMCH1* to metastatic progression in an independent cohort of invasive breast carcinoma (mixed subtypes), where *LIMCH1* protein expression was observed in 29.2% of tumors. In their retrospective cohort of 89 patients with invasive breast carcinoma, they performed IHC using the HPA004184 antibody (Atlas Antibodies, Bromma (Stockholm), Sweden, 1:1000 dilution). *LIMCH1* protein expression was associated with a higher frequency of lymph-node involvement and distant metastases. In multivariate Cox regression analysis, *LIMCH1* was identified as an independent adverse prognostic indicator for distant metastasis-free survival (DMFS; HR = 3.21); analysis with the KM-plotter database linked increased *LIMCH1* mRNA expression to poorer overall survival (HR = 2.73).

In a separate study, Yu et al. [5] connected high *LIMCH1* levels in breast cancer to enhanced glycolytic activity and nucleotide metabolism using transcriptomic correlation analyses, implicating a potential role in metabolic reprogramming; nonetheless, these findings are still correlational and require direct experimental confirmation. Complementing these transcriptomic data, Yu et al. [5] further validated *LIMCH1* protein overexpression by immunohistochemistry in an independent breast cancer cohort, linked high *LIMCH1* expression to an immunosuppressive infiltration pattern, and incorporated *LIMCH1* into a prognostic nomogram, providing protein-level and clinical support for a pro-tumorigenic role of *LIMCH1* in breast cancer. TGFβ signaling is known to reversibly toggle breast cancer cells between cohesive and single-cell migratory states [49], a phenotypic shift that may converge with *LIMCH1*-dependent cytoskeletal restructuring within the mechanically dynamic tumor microenvironment.

As detailed in Section 3.1, *LIMCH1*-dependent NM-IIA contractility governs the balance between mesenchymal and amoeboid migration modes. In the context of breast cancer metastasis, this mechanical switch may be particularly relevant: Holle et al. [50] showed that cancer cells confined in microchannels undergo mesenchymal-to-amoeboid transition solely by increasing Rho–ROCK contractility. If *LIMCH1* indeed tunes NM-IIA activity [1], its expression level may determine whether a cell adopts a mesenchymal (protease-dependent, slow) or amoeboid (contractility-driven, fast) migration mode in confined spaces such as lymphatic vessels or the interstitial matrix. In a recent drug screen [51], amoeboid melanoma cells—which rely heavily on actomyosin contractility—were selectively inhibited by specific small molecules that had negligible effects on mesenchymal variants. This pharmacological split suggests that the amoeboid state is a targetable vulnerability, and *LIMCH1*, as a regulator of NM-IIA contractility, may be a key determinant of whether cells enter this vulnerable state.

Further supporting a pro-tumorigenic role in breast cancer, Cizkova et al. [52] showed that *PIK3CA*-mutated, ERα-positive breast cancers exhibit elevated *LIMCH1* expression, suggesting a potential link between *LIMCH1* and PI3K/AKT-driven tumorigenesis.

### 4.3. Other Solid Malignancies: Preliminary Evidence

Gynecologic cancers provide additional support for a pro-tumorigenic role, albeit with heterogeneous evidence quality. Halle et al. [9] examined RNA-seq profiles from 73 primary cervical carcinomas that either later recurred or remained disease-free and derived a 10-gene prognostic signature that included *LIMCH1* and *HLA-DQB1*. Immunohistochemical assessment on a large tissue microarray *(n* = 410) confirmed that elevated *LIMCH1* protein expression, quantified by H-score, was an independent predictor of poorer disease-specific survival (HR = 3.19, *p* = 0.007) and correlated with nonsquamous histology and high tumor grade. The agreement between transcriptomic and protein-level findings in this cohort is notable, as many computationally generated signatures fail to be validated by IHC.

Beyond cervical cancer, bioinformatic studies have nominated *LIMCH1* as a candidate biomarker in several other malignancies, although experimental validation remains limited. In ovarian cancer, Kasavi et al. [11] used GEO-derived weighted gene co-expression network analysis (WGCNA) to identify *LIMCH1* as a novel biomarker; it clustered within a co-expression module containing *CRLS1*, *CDT1*, and *CNIH4*, but no functional assays were performed. In endometrial carcinoma, Bell et al. [12] and Le Gallo and Bell [13] identified *LIMCH1* as a significantly mutated gene within the hypermutated/microsatellite–unstable molecular subtype; in this context, a recurrent R421fs frameshift mutation truncates the C-terminal LIM domain, indicating that *LIMCH1* loss of function may promote endometrial tumorigenesis. In esophageal squamous cell carcinoma, Wen et al. [10] identified *LIMCH1* as one of three differentially expressed genes (together with *MMP1* and *C1orf226*) capable of predicting pathological complete response to neoadjuvant chemoradiotherapy. More recently, Xu et al. [14] included *LIMCH1* in a lysosome-dependent cell death-related molecular subtype framework for papillary thyroid carcinoma, where it helped to delineate three prognostic groups characterized by distinct immune signatures. These findings are exploratory and require mechanistic follow-up before firm conclusions can be drawn.

## 5. Methodological Drivers of Discordant Observations Across Studies

Beyond biological explanations, methodological factors substantially contribute to the apparent discrepancies among *LIMCH1* studies.

Antibody choice is a particularly underappreciated driver of variability. Both Cao et al. [2] and the TNBC-centered study by Qiu et al. [3] used the same Abcam (Cambridge, UK) ab96178 antibody but reached diametrically opposite conclusions regarding whether high or low *LIMCH1* expression predicts poor clinical outcomes. In contrast, Alifanov et al. [4] employed HPA004184 from Atlas Antibodies. Neither of these antibodies has been thoroughly validated for isoform specificity, and, given the existence of multiple *LIMCH1* splice variants [17,18], it is reasonable to hypothesize that different reagents may preferentially detect distinct subsets of isoforms with potentially divergent biological roles. For example, if ab96178 recognizes an epitope within the C-terminal LIM domain that is absent in certain truncated variants, tumors expressing predominantly truncated isoforms would be scored as falsely negative, whereas an antibody targeting the N-terminal CH domain might yield the opposite pattern. Systematic isoform-resolved antibody mapping is urgently needed to test this hypothesis.

Another frequently overlooked contributor to clinical inconsistency is the absence of harmonized IHC scoring methods. The criteria for defining *LIMCH1* positivity varied markedly between studies: Cao et al. [2] used a composite score based on staining intensity and the proportion of positive cells, Halle et al. [9] used a staining index (SI), whereas Alifanov et al. [4] adopted a binary threshold to classify protein expression. Such heterogeneous scoring schemes directly foster discordant prognostic associations, even when the same antibody is employed.

Analytical differences between processing pipelines add another source of quantitative variability. As outlined in Section 6, selecting the DESeq2 median-of-ratios workflow [53] versus FPKM/RPKM-based workflows can produce markedly different *LIMCH1* fold-change estimates within the same tumor entity. For example, in lung adenocarcinoma, the DESeq2 analysis detected no meaningful change in *LIMCH1* mRNA (log2FC = +0.02, *p* = 0.95), while earlier FPKM-based evaluations of TCGA data reported *LIMCH1* downregulation in this cancer type [2,3]. However, the extent of *LIMCH1* downregulation in clear-cell renal cell carcinoma was considerably greater in the DESeq2 pipeline (log2FC = −2.46) compared with prior FPKM-based estimates. These inconsistencies arise from analytical pipeline variation rather than genuine biological disagreement, highlighting the importance of standardized quantification approaches in upcoming multi-cohort investigations. Collectively, antibody isoform specificity, non-harmonized IHC scoring, and inconsistent quantification pipelines represent three interacting technical drivers of the conflicting prognostic associations reported across *LIMCH1* studies—and directly motivated the standardized analytical workflow applied in the pan-cancer analysis described in Section 6.

## 6. LIMCH1 Expression and Prognostic Value Across Human Cancers

### 6.1. Data Re-Analysis and Methodological Standardization

To eliminate systematic biases arising from heterogeneous RNA-seq quantification pipelines used in prior studies (FPKM/RPKM vs. raw counts), a unified re-analysis of raw STAR-Counts from TCGA was performed. All analyses were conducted in R (v4.2.3) using TCGAbiolinks (v2.25.3) to query the NCI GDC Data Portal (Data Release 36). Gene-level read counts were downloaded for primary tumors and matched solid-tissue normal samples across 19 cancer types with at least three normal specimens. Low-abundance genes were filtered by retaining only those with ≥10 total reads across all samples. Differential expression was assessed using DESeq2 (v1.38.0) with median-of-ratios normalization and negative-binomial generalized linear models (Wald test, Benjamini--Hochberg correction).

Overall survival (OS) was defined from the date of initial pathologic diagnosis to death or last follow-up. Patients were stratified into *LIMCH1*-high and *LIMCH1*-low groups by the median of raw *LIMCH1* counts within each cancer type. Univariate Cox proportional hazards models were fitted using the survival package (v3.4-0). Multivariate Cox models were constructed with stepwise covariate inclusion depending on data availability: the full model included age (continuous), pathologic stage (early [Stage I–II] versus advanced [Stage III–IV]), and sex; if the full model failed to converge, simplified models sequentially omitting covariates were attempted (age + sex, age alone, or stage alone). The proportional hazards assumption was verified using Schoenfeld residuals (survminer package). Hazard ratios (HR) and 95% confidence intervals (CI) are reported for the *LIMCH1*-high versus *LIMCH1*-low contrast.

### 6.2. LIMCH1 Expression and Prognostic Value

**Figure 1:**
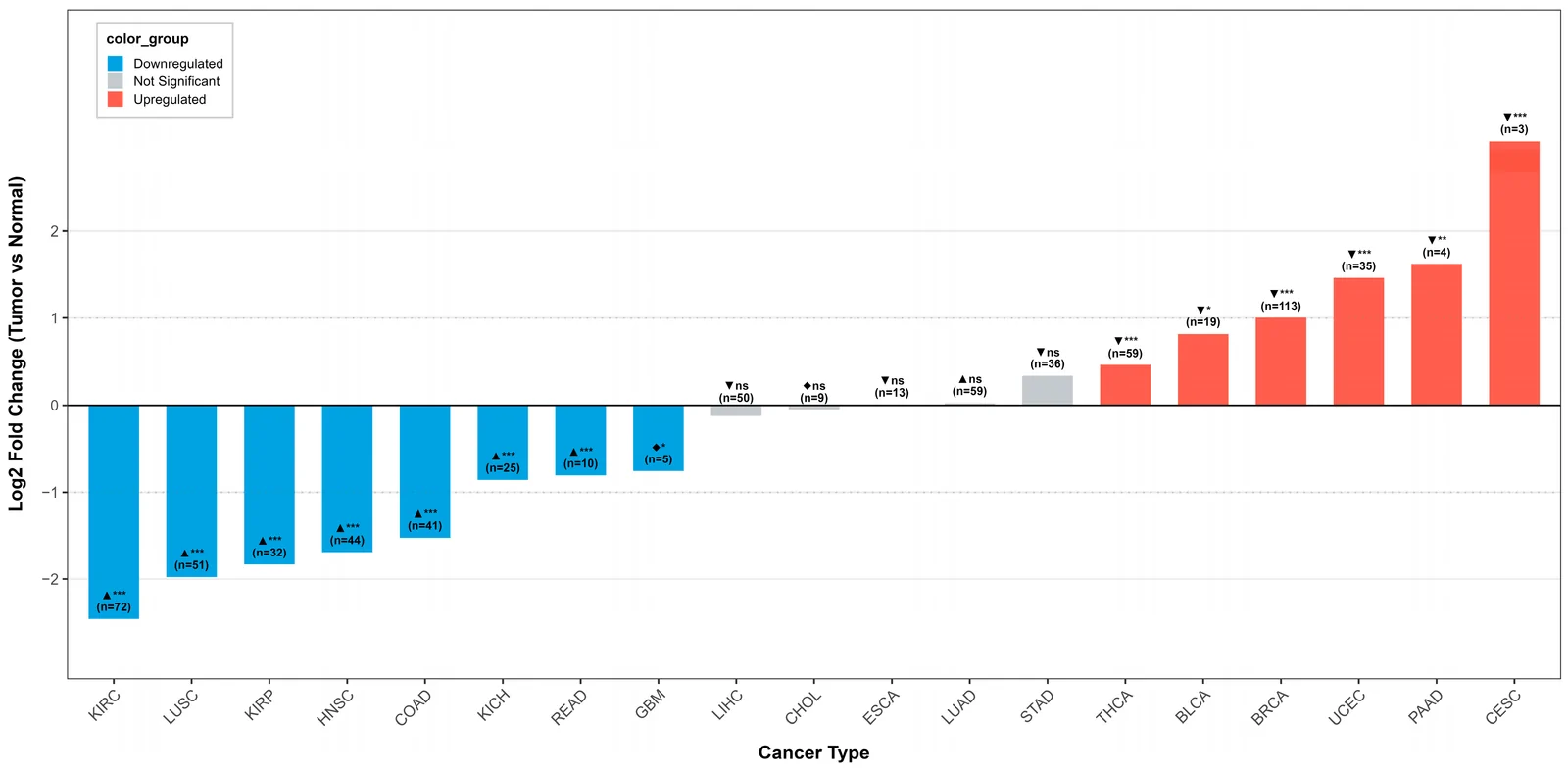
Pan-cancer profiling of *LIMCH1* expression. Gene expression data were extracted from The Cancer Genome Atlas (TCGA) using the TCGAbiolinks *R* package (v2.25.3; data category: Transcriptome Profiling; data type: Gene Expression Quantification; workflow: STAR–Counts; sample types: Primary Tumor and Solid Tissue Normal). Cancer types with at least three normal specimens (*n* = 19) were retained. Differential expression was assessed with DESeq2 (v1.38.0) using the design ~ sample type (tumor vs. normal), and *p*-values were corrected by the Benjamini–Hochberg method. Log_2_ fold changes of *LIMCH1* (*ENSG00000143375*) mRNA in tumor vs. normal tissues across 19 TCGA cancer types. Red: significant upregulation; blue: significant downregulation; gray: non-significant. ▲/▼/◆ = tumor-suppressive/pro-tumorigenic/bidirectional function reported in the literature; ns = not significant. * Adjusted *p* < 0.05; ** adjusted *p* < 0.01; *** adjusted *p* < 0.001. Numbers in parentheses = normal sample sizes. CESC (*n* = 3), PAAD (*n* = 4), and GBM (*n* = 5) had limited normal specimens; comparisons involving <10 normal samples should be interpreted cautiously owing to reduced statistical power. All analyses were performed in *R* v4.2.3.

*LIMCH1* mRNA levels differed markedly across tumor types (Figure 1). The strongest downregulation occurred in kidney renal clear-cell carcinoma (KIRC; log2 fold change = −2.46, adjusted *p* < 1.0 × 10^−15^), lung squamous cell carcinoma (LUSC; −1.98), kidney renal papillary cell carcinoma (KIRP; −1.83), head and neck squamous cell carcinoma (HNSC; −1.69), colon adenocarcinoma (COAD; −1.52), and kidney chromophobe (KICH; −0.86). Conversely, pronounced upregulation was observed in cervical squamous cell carcinoma (CESC; +3.03), pancreatic adenocarcinoma (PAAD; +1.62), uterine corpus endometrial carcinoma (UCEC; +1.46), breast invasive carcinoma (BRCA; +1.00), bladder urothelial carcinoma (BLCA; +0.82), and thyroid carcinoma (THCA; +0.46). No statistically significant alterations were identified in lung adenocarcinoma (LUAD; +0.02, *p* = 0.95), esophageal carcinoma (ESCA; +0.01), stomach adenocarcinoma (STAD; +0.34), liver hepatocellular carcinoma (LIHC; −0.13), or cholangiocarcinoma (CHOL; −0.05). Among the 19 cancer types analyzed, normal sample sizes ranged from 3 (CESC) to 72 (COAD), with a median of 24. 

This heterogeneous expression pattern provides a transcriptomic basis for reconciling previously conflicting functional findings in the literature. In cancers where *LIMCH1* is markedly upregulated, tumor cells appear to co-opt the protein into oncogenic signaling circuits—illustrated by its involvement in the MAPK–PD-L1 immune-regulatory axis in triple-negative breast cancer and the glycosyltransferase-associated subtype signatures in aggressive breast cancer forms. By contrast, in malignancies showing strong *LIMCH1* downregulation, the loss of this protein likely compromises cytoskeletal organization and removes a p53-stabilizing checkpoint, as observed in lung and renal cell carcinomas. The lack of clear differential expression in lung adenocarcinoma in this dataset stands in contrast to the reported tumor-suppressive role of *LIMCH1* in this cancer type; this inconsistency is most plausibly due to differences in quantification pipelines rather than genuine biological discordance (see Section 5). mRNA and protein levels are not always tightly correlated in clinical tumor specimens; post-transcriptional regulation can produce divergent protein expression, even when mRNA changes are minimal or absent. Therefore, the absence of significant mRNA-level differences in the DESeq2 results does not contradict the IHC-based evidence of reduced *LIMCH1* protein abundance in LUAD described by Cao et al. [2]. The pronounced downregulation in head and neck squamous cell carcinoma, which has not been emphasized in prior studies, constitutes a novel finding that merits additional exploration. In addition, the especially strong decrease in clear-cell renal cell carcinoma (log2FC = −2.46) surpasses earlier estimates from FPKM-normalized datasets, highlighting how quantitative interpretations depend critically on the chosen normalization approach.

**Figure 2:**
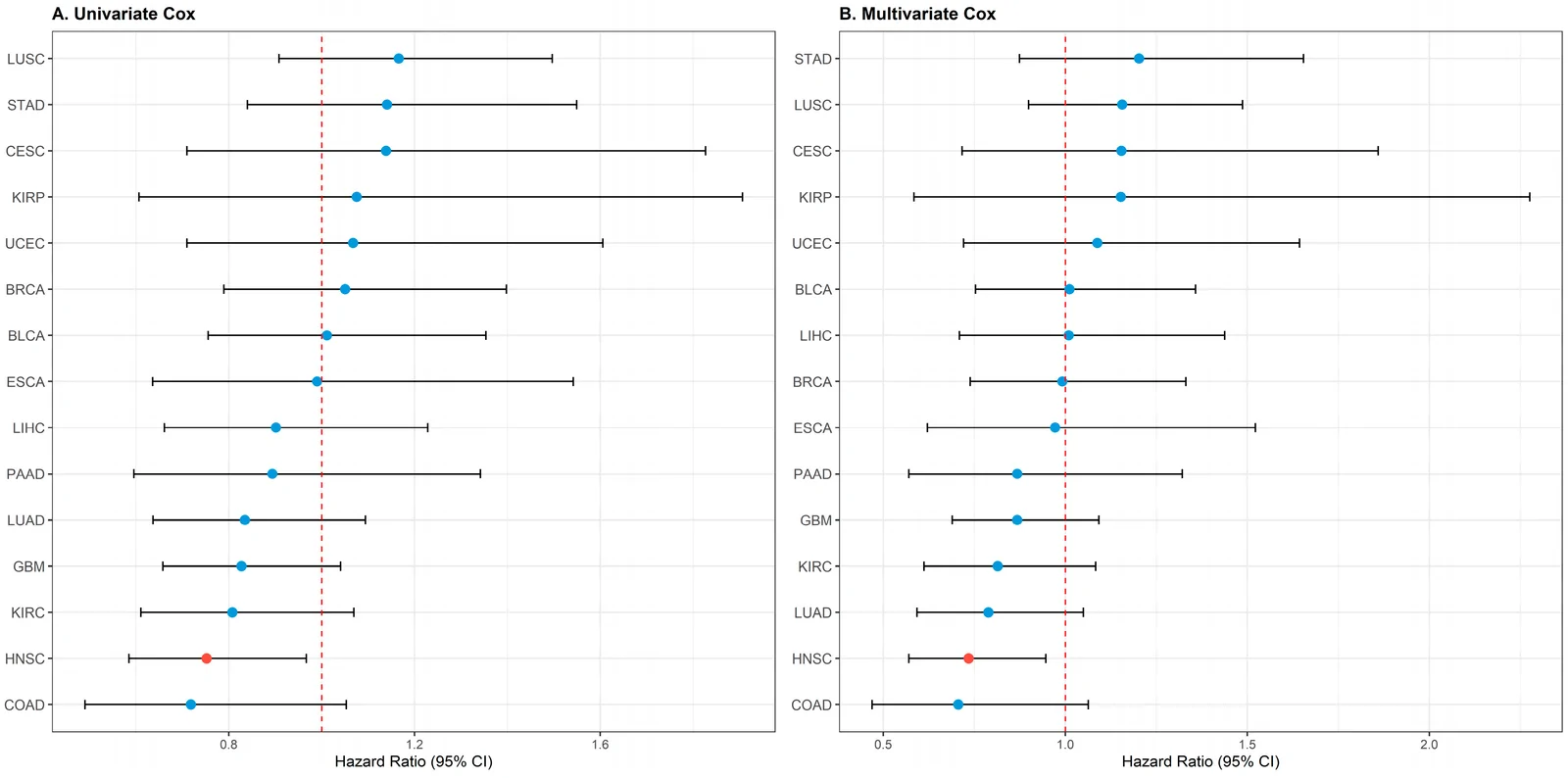
Prognostic value of *LIMCH1* mRNA expression across TCGA cancer types. Clinical data were retrieved from TCGA via the TCGAbiolinks *R* package (v2.25.3, GDCquery_clinic), and *LIMCH1* expression (STAR–Counts) was aggregated to the patient level using sample barcodes. Patients with overall survival time greater than 0 days were included, and *LIMCH1* expression was dichotomized into high versus low groups by the median value. (**A**) Univariate; and (**B**) multivariate Cox proportional hazards analyses (survival v3.4-0; survminer package) of overall survival comparing *LIMCH1*-high versus *LIMCH1*-low expression groups (median split). In the multivariate model, covariates were included in a stepwise manner depending on data availability: age, pathologic stage (early [Stage I–II] vs. advanced [Stage III–IV]), and sex. Hazard ratios (HR) and 95% confidence intervals (CI) are shown. Red dashed line, HR = 1; red dot, statistically significant (*p* < 0.05); blue dot, not significant (NS). In univariate analysis, elevated *LIMCH1* was significantly associated with better overall survival in HNSC (HR = 0.752, 95% CI 0.585–0.967, *p* = 0.026). After multivariate adjustment, the protective effect in HNSC remained significant (HR = 0.735, 95% CI 0.570–0.947, *p* = 0.017), whereas associations in colon adenocarcinoma (COAD) and lung adenocarcinoma (LUAD) did not reach statistical significance. All analyses were performed in *R* v4.2.3.

In the univariate Cox models (Figure 2A, elevated *LIMCH1* expression was significantly correlated with better overall survival in head and neck squamous cell carcinoma (HNSC; HR = 0.752, 95% CI 0.585–0.967, *p* = 0.026) and exhibited a trend toward a protective effect in colon adenocarcinoma (COAD; HR = 0.718, 95% CI 0.490–1.053, *p* = 0.090) and lung adenocarcinoma (LUAD; HR = 0.835, 95% CI 0.637–1.094, *p* = 0.191). No statistically significant univariate associations were observed in the remaining cancer types.

In multivariate Cox regression analyses adjusted for age, early versus advanced pathologic stage, and sex (Figure 2B), the protective effect in HNSC remained statistically significant (HR = 0.735, 95% CI 0.570–0.947, *p* = 0.017), indicating that *LIMCH1* serves as an independent favorable prognostic biomarker in this cancer type. Colon adenocarcinoma and lung adenocarcinoma showed effect estimates in the same direction as in the univariate analyses but did not achieve statistical significance after adjustment for covariates (COAD: HR = 0.706, 95% CI 0.469–1.063, *p* = 0.095; LUAD: HR = 0.789, 95% CI 0.593–1.049, *p* = 0.103). Kidney renal clear-cell carcinoma likewise showed a trend toward a protective effect (KIRC: HR = 0.814, 95% CI 0.612–1.083, *p* = 0.159). Critically, none of the tumor types displayed a significant association between higher *LIMCH1* expression and worse prognosis in the multivariate model.

## 7. Competing Molecular Circuits: A Context-Dependent Regulatory Model

**Figure 3:**
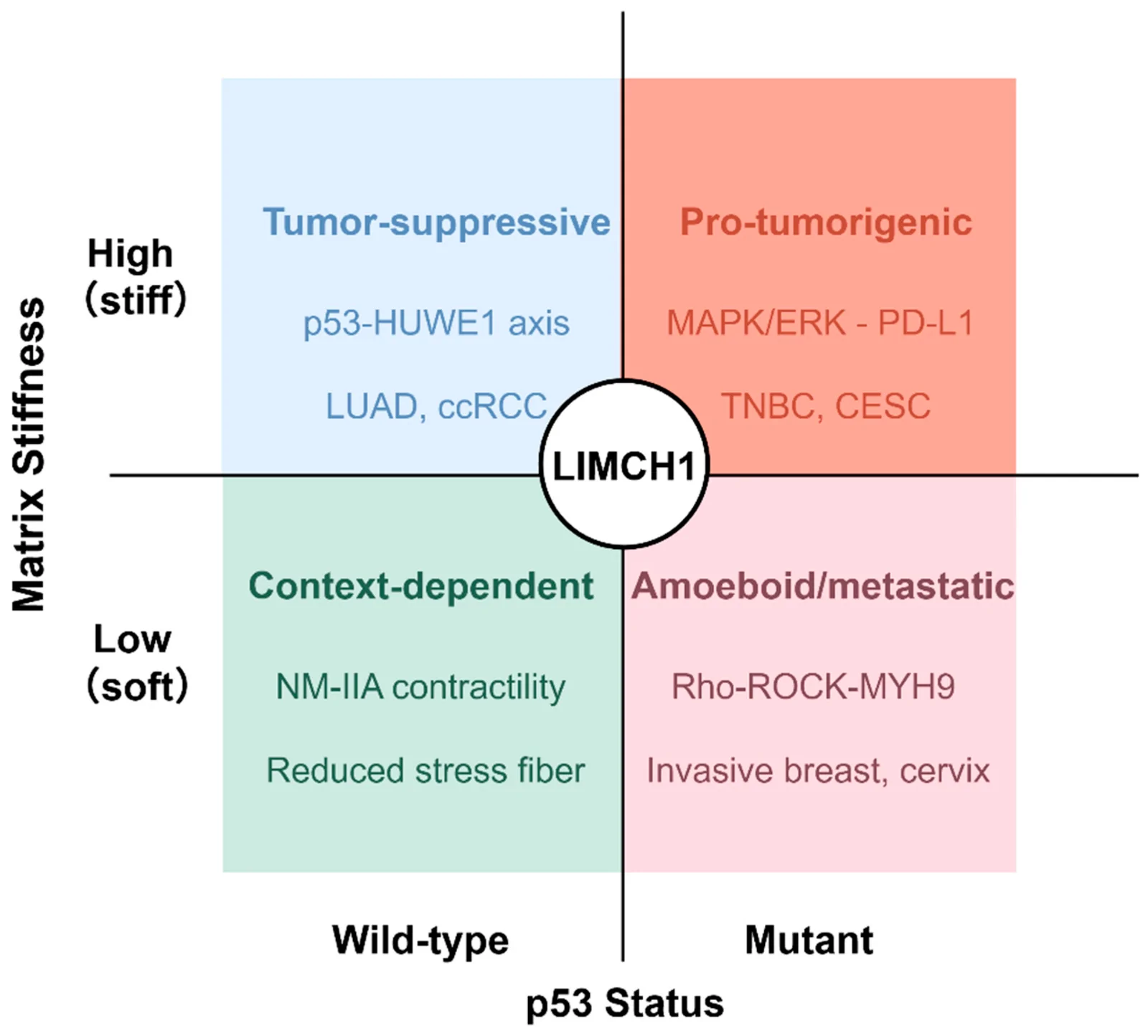
Context-dependent regulatory model of *LIMCH1*. The functional output of *LIMCH1* is governed by the intersection of *TP53* mutational status (*x*-axis) and tumor matrix stiffness (*y*-axis). In *TP53* wild-type, high-stiffness settings, *LIMCH1* exerts tumor-suppressive effects via the *HUWE1*–p53 axis (LUAD, ccRCC). In *TP53*-mutant, high-stiffness settings, *LIMCH1* promotes tumor progression via MAPK/ERK-PD-L1 signaling (TNBC, CESC). Under low-stiffness conditions, *LIMCH1* regulates NM-IIA (*MYH9*)-dependent contractility to influence the balance between mesenchymal and amoeboid migration modes. *X*-axis: *TP53* mutational status determines whether the *LIMCH1*-*HUWE1*-p53 axis is functional. *Y*-axis: matrix stiffness modulates NM-IIA contractility and migration mode (mesenchymal vs. amoeboid). The intersection of these two dimensions predicts *LIMCH1* functional output in different cancer contexts.

The seemingly opposing roles of *LIMCH1* in different cancers become coherent when three contextual factors are evaluated together: *TP53* mutation status; the biomechanical characteristics of the tumor microenvironment; and upstream signaling inputs. These axes converge to define *LIMCH1*’s net functional effect, as outlined in Figure 3.

We propose a hypothesis-generating conceptual framework (Figure 3), in which the functional output of *LIMCH1* is governed by the intersection of *TP53* mutational status (*x*-axis) and tumor matrix stiffness (*y*-axis). The *x*-axis is grounded in the experimental observation that *LIMCH1*’s interaction with *HUWE1* only protects p53 when p53 is structurally intact: in A549 lung adenocarcinoma cells (*TP53* wild-type), *LIMCH1* stabilizes p53 by blocking *HUWE1*, thereby limiting proliferation [16]. Under *TP53*-mutant conditions, as exemplified by the 4T1 TNBC model, this protective arm is abrogated, and *LIMCH1* instead activates the MAPK/ERK–PD-L1 circuit, facilitating immune evasion [3]. Therefore, *TP53* mutation status fundamentally alters the functional output of *LIMCH1* within this model. The *y*-axis is informed by the mechanobiology literature: because *LIMCH1* modulates NM-IIA–actin engagement [1,33,34], it affects whether cells undergo mesenchymal or amoeboid migration. In stiffer, fibrosis-like matrices (~10–30 kPa), loss of *LIMCH1* favors mesenchymal, protease-driven invasion, whereas in softer, confined channels, such as lymphatic vessels (~0.1–1 kPa), increased *LIMCH1* expression shifts cells toward amoeboid motility [34,35]. The third factor comprises upstream regulation—MAPK/ERK, TGFβ, and various non-coding RNAs all feed into *LIMCH1* expression [3,43,54], meaning that the signaling environment ultimately determines the abundance of *LIMCH1* available to execute the aforementioned functions.

This regulatory model resolves the apparent discordance between the pan-cancer survival analysis (Section 6) and the pro-oncogenic roles reported in breast and cervical cancers [3,4,9]. The TCGA multivariate analysis identified no significant adverse prognostic effect of high *LIMCH1* expression across tumor types because it did not stratify by *TP53* mutation status; the breast cancer dataset includes all molecular subtypes rather than TNBC alone. When *TP53* is wild-type, as in a subset of breast cancers, *LIMCH1* may still exert protective effects through the *HUWE1*-p53 axis; conversely, in *TP53*-mutant TNBC, the same protein promotes progression via MAPK-PD-L1 signaling. Similarly, cervical cancer and lung adenocarcinoma both show moderate-to-high immune infiltration; however, the predominant *LIMCH1*-regulated pathway—immunosuppressive in cervical cancer [9] versus tumor-suppressive in lung adenocarcinoma [2]—likely arises from differences in *TP53* status and the surrounding cytokine environment. It is critical to note that this framework is hypothesis-generating rather than an established mechanistic paradigm. The evidence hierarchy supporting this model includes: (i) expression and prognostic associations from clinical cohorts [2,3,4,9]; (ii) mechanistic evidence from cell-line experiments [1,3,16]; (iii) in vivo functional data from syngeneic mouse models [3]; and (iv) bioinformatic correlations from human clinical datasets (TCGA). Direct experimental evidence simultaneously manipulating TP53 status, matrix stiffness, and *LIMCH1* expression in isogenic systems is currently lacking; correlative findings—particularly from transcriptomic associations—should not be interpreted as causal mechanisms.

This regulatory model has defined boundary conditions worth noting. In hypermutated/microsatellite-unstable endometrial carcinoma, recurrent truncating mutations in *LIMCH1* (e.g., R421fs, indicating an arginine-421 frameshift mutation) lead to complete loss of the C-terminal LIM domain and abrogation of its physiological protein interactions [12,13]. Similarly, in cancer types where *LIMCH1* is transcriptionally silenced (e.g., by promoter hypermethylation), the protein is functionally absent. In both scenarios, *LIMCH1* loss-of-function drives tumorigenesis regardless of *TP53* status or matrix stiffness. The context-dependent model, therefore, applies primarily to tumors with an intact *LIMCH1* expression and protein structure, rather than those with complete loss of *LIMCH1* via genetic or epigenetic mechanisms. This distinction is clinically relevant, as the latter may require a different therapeutic strategy (e.g., restoration of *LIMCH1* expression) compared to the former.

**Figure 4:**
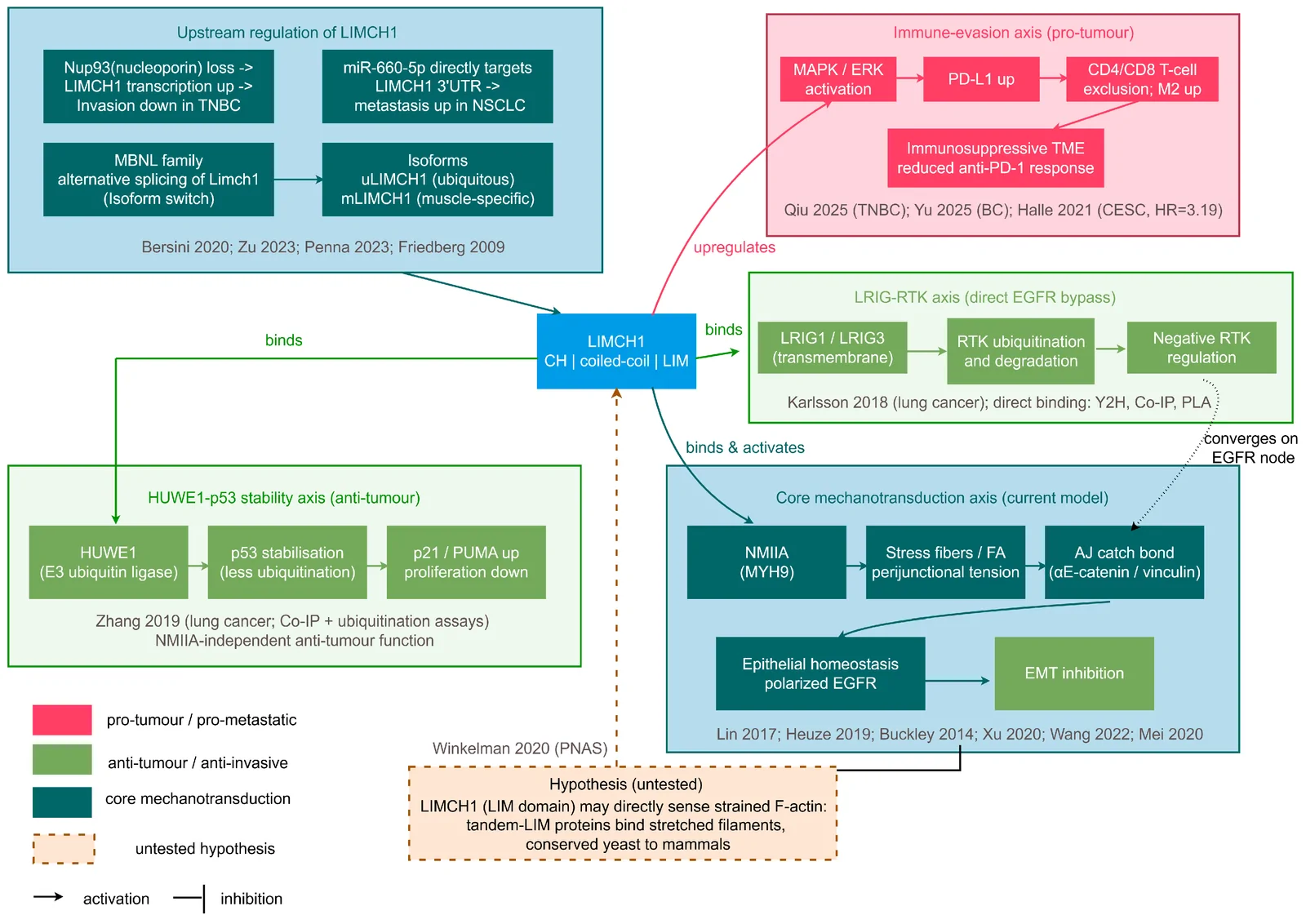
*LIMCH1*-centered regulatory network in cancer. *LIMCH1* (blue hub; CH, coiled-coil and LIM domains) integrates five experimentally supported modules: (i) core mechanotransduction (slate)—*LIMCH1* binds and activates NM-IIA (*MYH9*) [1], sustaining stress fibers, focal adhesions and perijunctional tension; (ii) immune evasion (red)—*LIMCH1* upregulates MAPK/ERK signaling and PD-L1, driving CD4/CD8 T-cell exclusion and M2-macrophage enrichment [3,5,9]; (iii) LRIG–RTK axis (ochre)—direct binding to LRIG1/LRIG3 links *LIMCH1* to RTK ubiquitination [38]; (iv) *HUWE1*–p53 stability axis (green)—*LIMCH1* binds *HUWE1*, reducing p53 ubiquitination and promoting p21/PUMA-mediated growth arrest [16]; and (v) upstream regulation (slate)—*NUP93* (transcription) [37], *miR-660-5p* (3′-UTR targeting) [44] and MBNL-dependent alternative splicing [17,18]. The gold dashed module denotes the untested hypothesis that the LIM domain of *LIMCH1* directly senses strained F-actin [55]. Arrows, activation; flat heads, inhibition; dashed lines, hypothesis. Author–year citations shown in the figure correspond to the following references: Lin et al. 2017 [1]; Zhang et al. 2019 [16]; Friedberg 2009 [17]; Penna et al. 2023 [18]; Heuze et al. 2019 [25]; Buckley et al. 2014 [27]; Xu et al. 2020 [28]; Wang et al. 2022 [29]; Mei et al. 2020 [30]; Bersini et al. 2020 [37]; Karlsson et al. 2018 [38]; Zu et al. 2023 [44]; Halle et al. 2021 [9]; Qiu et al. 2025 [3]; Yu et al. 2025 [5]; and Winkelman et al. 2020 [55].

These three dimensions are not independent; they intersect and modulate one another. For example, though both lung adenocarcinoma and TNBC exhibit high baseline *TP53* mutation frequencies, the distinct mechanical contexts of lung parenchyma and breast stroma may bias cells toward different *LIMCH1*-dependent migration programs. The full *LIMCH1*-centered regulatory network integrating these axes is summarized in Figure 4.

## 8. Clinical Implications and Prognostic Value

From a translational standpoint, the key question is whether *LIMCH1* can serve as a reliable biomarker. The most plausible near-term scenarios are not pan-cancer screening but context-specific adjuncts: postoperative recurrence stratification in lung adenocarcinoma, and immunotherapy-response enrichment in TNBC when *LIMCH1* is interpreted together with PD-L1 and immune-cell exclusion features. Before clinical use, the field needs prespecified cutoffs—the median expression, H-score or staining index should be validated against one harmonized reference assay rather than carried over between studies—plus standardized antibody clones, scoring training, and blinded endpoint review. Biologically rational combinations include *LIMCH1* with PD-L1 and T-cell markers for immune exclusion, with p53 IHC or *TP53* mutation for the *HUWE1* axis, and with stiffness/EMT features for migration phenotype. The dominant bottleneck remains the absence of prospective, multicenter cohorts with predefined thresholds and clinically meaningful endpoints.

The clinical scenario in breast cancer is particularly complex. If *LIMCH1* indeed enhances PD-L1 expression through MAPK/ERK signaling, it may hold promise as a candidate biomarker for anti-PD-1 therapy, but in an unconventional way: elevated *LIMCH1* would be predicted to indicate not heightened sensitivity to immunotherapy—as is usually inferred from PD-L1–based markers—but rather an immunosuppressive tumor milieu in which checkpoint blockade is unlikely to work. We emphasize, however, that *LIMCH1* currently remains a candidate biomarker requiring prospective clinical validation in multicenter cohorts with predefined thresholds. Qiu et al. [3] provided preliminary in silico evidence for this hypothesis by associating high *LIMCH1* expression with poor response to anti-PD-1 treatment; however, this has not been corroborated by clinical trial data. Conversely, in lung and renal cancers, where *LIMCH1* appears to exert a protective role, approaches focused on restoring its expression could be justified, although no druggable domain within *LIMCH1* has been identified and its protein structure remains unresolved.

Prognostic modeling likewise demands careful treatment of covariates. Terrematte et al. [8] incorporated *LIMCH1* into a 13-gene prognostic signature for renal cancer, while Zu et al. [44] included it in a six-gene NSCLC metastasis predictor. In both frameworks, *LIMCH1* improved multivariate prognostic performance; however, the biological plausibility and internal consistency of these composite gene panels remain uncertain. Machine learning methods can generate statistically convincing gene sets that lack any unifying mechanism and there is an ongoing risk of overfitting, especially when models are trained on relatively small, publicly accessible datasets.

The prognostic relevance of *LIMCH1* does not map in a simple, linear fashion onto baseline *TP53* mutation frequencies or the overall degree of immune infiltration across tumor types. For instance, lung adenocarcinoma and triple-negative breast cancer both harbor high baseline *TP53* mutation rates, yet increased *LIMCH1* expression correlates with opposite clinical outcomes in these cancers. Similarly, cervical cancer and lung adenocarcinoma each exhibit moderate-to-high immune cell infiltration, but *LIMCH1* conveys different prognostic meanings in these contexts. Overall, these findings indicate that *LIMCH1*’s prognostic value cannot be attributed to a single molecular pathway; rather, it appears to emerge from the interaction of multiple context-dependent factors.

By contrast, the contribution of *LIMCH1* to predicting treatment response has been relatively less studied. Wang et al. [43] showed that *circLDB2*-driven upregulation of *LIMCH1* enhances cisplatin sensitivity in NSCLC. Wen et al. [10] identified *LIMCH1* as a marker of pathological complete response to neoadjuvant chemoradiotherapy in esophageal squamous cell carcinoma. These observations imply that *LIMCH1* status may shape therapeutic responsiveness in addition to its prognostic role, although prospective confirmation is still missing. Future studies should consider post hoc analyses of existing immunotherapy cohorts—such as IMvigor210, the KEYNOTE trials, or comparable OAK/POPLAR datasets—to determine whether *LIMCH1* expression stratifies responses to immune checkpoint inhibitors.

### Limitations of the Evidence Base and of This Review

Several limitations qualify the conclusions above. Most clinical studies are retrospective and single-center, and even supportive cohorts rarely validate the same cutoff in an independent prospective population. Mechanistic claims rest heavily on a small set of systems—A549 and related lung models, 4T1 and breast-cell models, and engineered overexpression or knockdown contexts—thus, it remains uncertain which observations generalize across tumor lineages. The pan-cancer re-analysis inherits the composition of public datasets, including uneven normal-tissue availability, batch effects and incomplete clinical covariates; apparent prognostic associations can, therefore, shift after adjustment. Finally, causal inference is limited by the lack of in vivo, tissue-specific genetic manipulation or *LIMCH1*-selective pharmacology; publication bias may favor reports in which high or low expression is framed as prognostically meaningful.

This review also has limitations. It is a structured narrative review rather than a formal systematic review; although the search strategy is described, study selection was guided by mechanistic relevance and may under-represent negative or neutral findings. Non-English literature, unpublished datasets, and preprints were not systematically captured. These constraints argue for treating the integrated model as a hypothesis-generating synthesis, not as clinical guidance.

## 9. Conclusions and Future Perspectives

### 9.1. Open Questions and Controversies

Five questions remain unresolved. First, when *LIMCH1* mRNA is unchanged, but protein is reduced, as reported in lung cancer, the responsible post-transcriptional mechanism—miRNA control, isoform switching, translation efficiency or protein stability—has not been defined. Second, the molecular switch that converts *LIMCH1* from tumor-suppressive to pro-tumorigenic is still unsettled: *TP53* mutation is a strong contextual candidate but is unlikely to be the sole determinant. Other factors—including isoform-specific effects, tissue-of-origin epigenetic programs, stromal stiffness, and additional tumor-lineage and microenvironmental cues—may act as contextual modulators that remain to be systematically tested. Third, the expression and function of *uLIMCH1* versus *mLIMCH1* isoforms across cancer types are almost unexplored. Fourth, possible cell-autonomous roles of *LIMCH1* in immune cells, including T cells and macrophages, have not been tested, even though immune-evasion phenotypes are increasingly linked to tumor-cell *LIMCH1*. Fifth, the hypothesis that the LIM domain directly senses mechanically stressed F-actin remains attractive but unproven in *LIMCH1* itself.

### 9.2. Experimental Roadmap for Validating the Context-Dependent Model

To facilitate validation of the context-dependent model proposed here, we suggest the following concrete research approaches:

1. p53-stratified cohort analysis: Existing TCGA and independent clinical cohorts should be re-analyzed for *LIMCH1* expression and survival after stratifying by *TP53* mutation status, ideally with subtyping of breast cancer (TNBC vs. non-TNBC). This would provide immediate retrospective evidence for the model’s central prediction;

2. Isoform-resolved cancer profiling: Using long-read sequencing (e.g., PacBio or Oxford Nanopore) together with isoform-specific quantitative PCR, a systematic map of *LIMCH1* splice variants across major cancer types should be established. This would determine whether isoform switching underlies the context-dependent functions attributed to *LIMCH1*. Indeed, this is the most pressing of all priorities: isoform-specific antibodies and targeted sequencing are needed to map which variants actually dominate in individual cancer types, and to determine whether isoform-restricted functions explain the divergent phenotypes reported in the literature;

3. In vivo matrix stiffness modulation: Using genetically engineered mouse models of lung or breast cancer combined with pharmacological modulation of collagen crosslinking (e.g., lysyl oxidase inhibitors), the role of matrix stiffness in dictating *LIMCH1*’s functional output could be directly tested.

It is equally important to note that the evidence for a tumor-suppressive role of *LIMCH1* in lung and renal cancers remains largely correlational—germline or conditional knockout models in autochthonous tumor settings are still required to rigorously test causality in vivo.

### 9.3. Therapeutic Outlook

Reducing *LIMCH1* to a simple designation as either a tumor suppressor or an oncogene neglects the biological complexity revealed by numerous studies. *LIMCH1* functions at the crossroads of cytoskeletal organization, nuclear signaling, immune modulation, and metabolic regulation—each of which is strongly shaped by cellular context. The body of evidence summarized here, together with the pan-cancer bioinformatic analyses, suggest that *LIMCH1*’s seemingly opposing roles arise from context-dependent consequences rather than a true mechanistic contradiction. We propose that its behavior appears to be primarily dictated by three factors: the mutational landscape of the tumor cell; the biomechanical characteristics of the surrounding microenvironment (including substrate rigidity and spatial confinement); and upstream signaling cues (such as MAPK/ERK and TGFβ pathways). In p53-competent lung cancer cells, *LIMCH1* promotes p53 stabilization by sequestering *HUWE1* and reinforcing NM-IIA-mediated contractility; in contrast, in p53-mutant TNBC, it intensifies MAPK-driven PD-L1 upregulation and supports an immunosuppressive milieu.

This re-analysis identified methodological discrepancies: the apparent magnitude—and even direction—of *LIMCH1* differential expression shifts noticeably, depending on the bioinformatic workflow. When raw STAR-Counts were processed through DESeq2’s median-of-ratios normalization, the fold changes differed from earlier studies that relied on FPKM matrices and parametric t-tests. These methodological divergences, rather than biological disagreement, probably account for some of the numerical inconsistencies across publications. Any comparison of *LIMCH1* expression levels should, therefore, explicitly note whether the data were generated via count-based negative binomial models or length-normalized intensity pipelines.

On the translational front, clinical implementation of *LIMCH1*-based biomarkers will hinge on standardized detection workflows. These must include antibody cross-validation, isoform-aware RNA-seq quantification pipelines, and uniform normalization schemes—the very methodological harmonization whose absence has fueled many of the conflicting reports discussed throughout this review. Post hoc analyses of completed immunotherapy cohorts (e.g., IMvigor210, KEYNOTE, or OAK/POPLAR) would also help to clarify whether *LIMCH1* expression can stratify patients for immune checkpoint inhibitor response. Looking further ahead, genetically engineered mouse models with tissue-specific *LIMCH1* knockout or overexpression will be critical for testing the context-dependent framework proposed here. Advances in spatial transcriptomics, single-cell proteomics, and live-cell biosensor imaging are likely to soon provide the resolution required to decipher the contextual “grammar” that shapes *LIMCH1*’s functional outcomes.

Until then, *LIMCH1* is best conceptualized as a conditional regulator—tumor-suppressive in certain malignancies, permissive in others—and should never be evaluated apart from the molecular and mechanical landscape of the tumor. In the near term, its most realistic translational application lies not in direct pharmacological targeting (no druggable domain has yet been identified), but in companion diagnostic development: stratifying patients for immunotherapy enrichment; predicting recurrence risk after surgical resection; and guiding the selection of mechanistically rational combination regimens. Future work should also clarify the role of *LIMCH1* in cancer-associated fibroblasts, as these cells are key drivers of tumor matrix stiffness and may transmit non cell-autonomous influences of *LIMCH1* on tumor progression.

## Data Availability

No data were used for the research described in the article.

## Abbreviations

*LIMCH1*, LIM and calponin homology domain-containing protein 1; NM-IIA, non-muscle myosin-IIA; FAK, focal adhesion kinase; MRLC, myosin regulatory light chain; TNBC, triple-negative breast cancer; ccRCC, clear-cell renal cell carcinoma; NSCLC, non-small cell lung cancer; MAT, mesenchymal-to-amoeboid transition; EMT, epithelial-to-mesenchymal transition; TCGA, The Cancer Genome Atlas; GTEx, Genotype-Tissue Expression; GSEA, gene set enrichment analysis; IHC, immunohistochemistry; HR, hazard ratio; CI, confidence interval; PD-L1, programmed death-ligand 1; ceRNA, competing endogenous RNA; GWAS, genome-wide association study; WGCNA, weighted gene co-expression network analysis; SNP, single nucleotide polymorphism; PCP, planar cell polarity; GTRGs, glycosyltransferase-related genes; CAF, cancer-associated fibroblast; LUAD, lung adenocarcinoma; LUSC, lung squamous cell carcinoma; KIRC, kidney renal clear-cell carcinoma; BRCA, breast invasive carcinoma; CESC, cervical squamous cell carcinoma; OV, ovarian serous cystadenocarcinoma; UCEC, uterine corpus endometrial carcinoma; PAAD, pancreatic adenocarcinoma; SKCM, skin cutaneous melanoma; COAD, colon adenocarcinoma.
